# The Impact of Intensified Hemodialysis on Pruritus in an End Stage Renal Disease Patient with Biliary Ductopenia

**DOI:** 10.1155/2015/236419

**Published:** 2015-03-04

**Authors:** Sandra Chomicki, Omar Dahmani

**Affiliations:** ^1^Service de Néphrologie, Centre Hospitalier Louis Pasteur, 4 rue Claude Bernard, 28 630 Le Coudray, France; ^2^Service de Néphrologie, Centre Hospitalier Louis Jaillon, 2 rue Hôpital, 39 206 Saint-Claude, France

## Abstract

We report a unique observation characterized by the coexistence of idiopathic adulthood ductopenia (IAD), a rare cholestatic disease, and end stage renal failure treated by conventional hemodialysis in a patient awaiting double renal and liver transplantation. As pruritus gradually worsened, we hypothesized that intensified dialysis could alleviate the symptoms. Conventional hemodialysis following 3 hours/3 times a week regimen was initiated in December 2013. Due to increasing pruritus not responding to standard medical therapy, intensified hemodialysis following 2.5 hours/5 times a week regimen was started in May 2014. During two weeks, a temporary decrease in bilirubin levels was observed. No major changes on other liver function tests and inflammatory markers occurred. Nevertheless, a persistent improvement on pruritus and general wellbeing was obtained during the four weeks' study period. The pathogenesis of itch encompasses multiple factors, and, in our case, both uremic and cholestatic pruritus are involved, although the latter is likely to account for a greater proportion. By improving itch intensity, through better clearance of uremic and cholestatic toxins which we detail further, intensive dialysis appears to be an acceptable short-term method for patients with hepatic cholestasis and moderate pruritus not responding to conventional therapy. Additional studies are needed to assess and differentiate precisely factors contributing to pruritus of both origins.

## 1. Introduction

Idiopathic adulthood ductopenia (IAD) is a rare cholestatic liver disease of unknown etiology first described by Ludwig et al. in 1988 [1]. To date, less than a hundred cases have been described in the literature. It is characterized by adult onset, biochemical evidence of cholestatic liver disease, negative antibodies, absence of bowel inflammatory disease, normal cholangiography, and loss of interlobular bile ducts on liver biopsy. The diagnosis requires exclusion of other conditions of chronic cholestasis. Interestingly, Li et al. [2] found an excellent correlation between the histologic classification and the clinical diagnosis. The course of the disease varies, severe IAD types typically progressing to end stage liver disease and requiring transplantation [3–5].

## 2. Case Presentation

**Figure 1:**
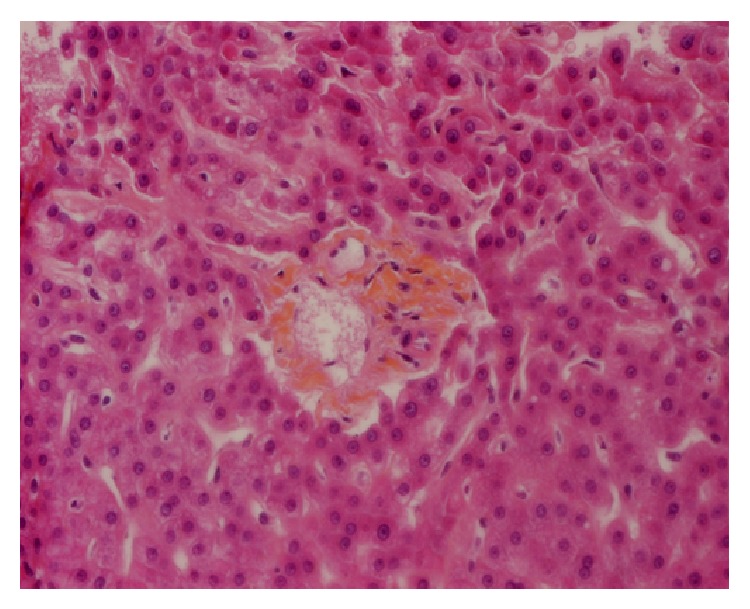
The liver biopsy specimen (H&E, ×200) shows branches of the hepatic artery and portal vein, but absence of interlobular bile duct. Lymphocyte infiltrate, cholangiolar proliferation, and fibrosis are absent.

We report the case of a 55-year-old patient presenting with a typical idiopathic adulthood ductopenia diagnosed earlier in the course of chronic renal failure requiring hemodialysis, awaiting double liver and renal transplantation. She was referred in 2008 to our clinic for impaired renal function and a biological cholestatic profile. Medical history included moderate hypertension and hysterectomy for fibroids causing metrorrhagia. There had been no family history of renal or liver disease and no personal history of recurrent urinary tract infection, medication treatments, illicit drug, or alcohol use. Physical examination showed moderate hypertension, mild jaundice, and mild pruritus. No clinical or biological signs of liver failure or encephalopathy were present. Laboratory tests revealed serum creatinine levels of 204 *μ*mol/L, hemoglobin 13.1 g/dL, calcium 2.48 mmol/L, phosphorus 1.07 mmol/L, elevation of transaminases three times the upper limit (AST 156 UI/L and ALT 182 UI/L), gamma-glutamyl transferase (GGT) 1000 UI/L, and ALP 1029 UI/L. Bilirubin levels were normal. Thyroid-stimulating hormone levels, urinary electrolytes, iron studies, serum immunoglobulins, and protein electrophoresis were normal. Hepatitis and autoimmune antibodies were negative. Proteinuria was 0.24 g/24 h. Renal ultrasound showed bilateral small kidneys (7 and 8 cm) with no signs of obstruction; abdominal ultrasound showed a normal liver size (10.5 cm), smooth contours, and no signs of biliary dilation. Renal biopsy was not performed due to kidneys' small size and a chronic renal failure profile. The diagnosis of IAD was obtained on a second liver biopsy in 2012, showing loss of interlobular bile ducts, no lymphocyte inflammatory infiltrate, no fibrosis, and no cholangiolar proliferation (Figure 1). Biological follow-up showed development of secondary hyperparathyroidism (parathyroid hormone (PTH) 539 pg/mL, calcium 1.82 mmol/L, and phosphorus 2.35 mmol/L in December 2013), stabilisation of transaminase levels around three times the upper fold, a decrease of GGT levels (226 UI/L in December 2013), and a steady increase of bilirubin levels (total bilirubin 144 *μ*mol/L, conjugated bilirubin 129 *μ*mol/L in December 2013). Along with emollients, pruritus was treated first with cholestyramine and second with ursodeoxycholic acid; however neither showed a significant improvement. Conventional dialysis was started in December 2013, five years after the initial diagnosis of chronic renal failure. The patient was registered for double renal and liver transplantation shortly after. As the liver disease progressed, with jaundice and pruritus gradually worsening, we decided in May 2014 to perform intensified dialysis in order to assess whether this regimen could alleviate the symptoms.

Conventional hemodialysis following a 3 hours/3 times a week regimen was initiated in December 2013, switched on May 27, 2014, for intensified hemodialysis following 2.5 hours/5 times a week regimen. Both were performed as high-flux hemodialysis and did not differ in terms of ultrapure water quality, dialysis machine (5008, Fresenius Medical Care), heparin use, vascular access (jugular dialysis catheter), high-flux dialysis filter (FX 80), surface (1.8 m^2^), blood flow (300 mL/min), and dialysate flow (500 mL/min). Mean *Kt*/*V* did not differ much ranging between 1.3 and 1.4. MARS and SPAD were not available at our centre and as for plasmapheresis, reports in these situations are anecdotal. At commencement of this regimen, no symptoms and signs of hepatic encephalopathy or liver failure were present with TP being 74% and serum albumin being 31.7 g/L.

**Figure 2:**
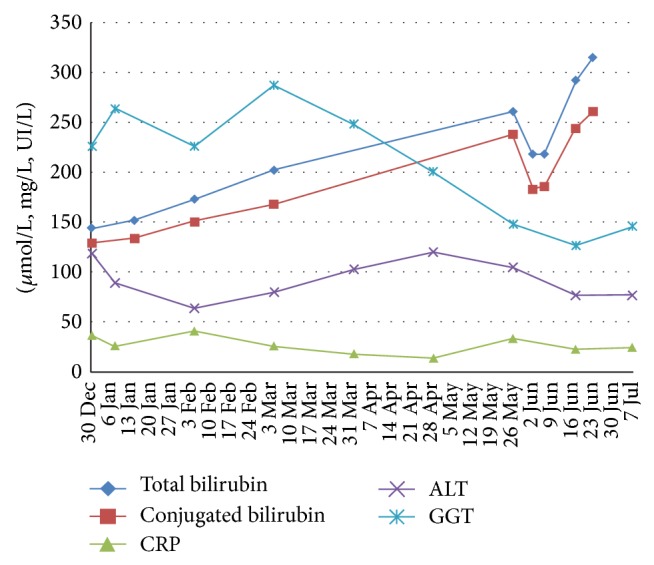
The graph shows a temporary drop in conjugated bilirubin levels after the initiation of intensified dialysis (May27, 2014), followed by a consistent increase. No other changes of Liver Function Tests or CRP were observed.

Initially, during an approximate two-week period, a decrease in bilirubin levels was observed (conjugated bilirubin levels were 238 *μ*mol/L on May 27, 186 *μ*mol/L on June 7), however followed by a continuous increase later on (261 *μ*mol/L on June 24). There were no major changes on other liver enzymes including ASP, ALT, GGT, and ALP and inflammatory markers including CRP and ferritin. PTH levels did not decrease after implementation of this regimen (560 pg/mL on July 8 versus 358 pg/mL in April 2014) and predialysis calcemia and phosphorus levels remained overall stable after intensified dialysis (resp., 1.92 mmol/L and 3.08 mmol/L on June 24 versus 1.94 mmol/L and 2.67 mmol/L on May 27). Taking a glance at the bilirubin curve, intensified dialysis did not change its increasing course (Figure 2). Nevertheless, a persistent improvement on pruritus was obtained over the four-week period we applied this regimen, with itch intensity being reduced by half. As this study was performed on one patient, a numeric assessment such as the visual analogue scale was not used as considered too subjective. Another weighty point in favor of this scheme is the pruritus rising on nondialysis days, thus showing a degree of effectiveness from hemodialysis on this symptom. Therefore we decided to continue this regimen until transplantation, as the improvement on pruritus and general wellbeing outweighed the inconvenience caused by daily travels.

## 3. Discussion

Pruritus is a common symptom in cholestatic disorders, occurring in about 30 to 70% patients with cholestatic liver disease according to available data. The pathogenesis of cholestatic pruritus is not clear. Some studies suggest that the accumulation of bile salts in the plasma and tissues of patients with cholestatic disease leads to pruritus, while some others propose that endogenous opioids play a key role in the development of pruritus [6, 7]. The latest studies have however focused on lysophosphatidic acid (LPA, monoacylglycerol-3-phosphate, <3 kDalton, and *t*_1/2_ = a few minutes) a water soluble phospholipid that could possibly act as a neuronal activator. In recent studies by Kremer et al. [8, 9], LPA acted as a major Ca^2+^ agonist in pruritic sera and its concentration was increased in patients with cholestatic pruritus. The presence of autotaxin (ATX, >100 kDalton), the enzyme that converts lysophosphatidylcholine into LPA, was also increased in these patients. Its activity was associated with cholestatic pruritus and correlated with the intensity of pruritus, which was not the case for serum bile salts, histamine, tryptase, substance P, or *μ* opioids. Additionally, Rifampicin, MARS treatment, and nasobiliary drainage all markedly reduced ATX serum levels, whereas ATX protein was neither directly drained into bile nor removed in albumin dialysate (MARS membrane pores having a molecular weight cutoff of 50 kDalton). The authors hypothesized that a factor capable of increasing ATX expression (or reducing its clearance) was removed by these treatments. Moreover, Beuers et al. [10] suggested that some inflammatory cytokines could contribute to increasing ATX levels during cholestasis. In light of these recent results, we could imagine that some small to medium molecular weight toxins responsible for pruritus could be partially removed by hemodialysis.

It would be very tempting to assume that the renal dysfunction in our patient was secondary to the liver condition. Recently, van Slambrouck et al. [11] carried out a remarkable study on bile cast nephropathy, a histologic entity observed in the spectrum of cholestatic pruritus. Following their results, a good correlation was observed between patients with hepatorenal syndrome and the presence of bile casts (85%) but poor in patients whose initial condition was cholestatic/obstructive jaundice (43%). Moreover, the presence of bile cast nephropathy is correlated with high bilirubin levels, while, on presentation, our patient's bilirubin levels were normal. Therefore, we cannot conclude here on a clear link between these two conditions.

Concerning intensified dialysis, this method has been the subject of growing interest for the past few years as conventional regimens (usually 4-hour sessions 3 times a week) did not offer optimal physiological replacement of renal function. Increasing dialysis time and/or allowing a more physiological renal replacement through daily sessions could offer better results. Intensified dialysis usually consists in short daily sessions, lasting 2 or 3 hours, or nocturnal 6- to 8-hour intermittent sessions 3 to 7 times a week. Intensified dialysis has shown in several previous studies improved outcomes on various measures, including arterial blood pressure, left ventricular hypertrophy, uremia associated variables, erythropoiesis parameters, and necessity of dietary restriction compared to conventional dialysis [12, 13]. A positive impact on inflammation has been suggested, as CRP tended to decline, although this has not been the case in our study.

We aimed to assess whether intensified hemodialysis could relieve cholestatic pruritus not responding to standard medical therapy. It is now generally accepted that liver dialysis devices such as molecular adsorbents recirculation system (MARS) and single pass albumin dialysis (SPAD) are effective in the management of intractable cholestatic pruritus as they remove a number of toxins including ammonia, albumin-bound bilirubin, bile acids, and phenols. Their use is currently a new emerging indication for severe pruritus [14–16].

However, these devices are not available everywhere and paramedical staff is not always trained. Moreover, our patient expressed moderate pruritus and no signs of liver failure or encephalopathy were present.

From our experience, intensified dialysis appears to be an acceptable short-term method (in our case, until transplantation) for moderate pruritus in patients affected with end stage renal disease and hepatic cholestasis not responding to conventional therapy. The pathogenesis of pruritus involves multiple factors. In our observation, cholestatic pruritus is likely to account for a greater proportion than uremic pruritus, as itch slowly but constantly increased over months, which correlates well with the natural course of the liver disease. Moreover, little benefit was achieved on parameters such as phosphorus, PTH, and calcium, thus suggesting a less important influence of uremic pruritus on itch [17]. Unfortunately, we did not measure autotaxin activity, which could have been a potential marker to differentiate its origin. Our case report also supports the hypothesis that other molecules, for instance, inflammatory cytokines, could play a role in the generation of cholestatic pruritus [10], as improving their clearance through intensive dialysis decreases itch intensity.

In this regard, studies including hemodiafiltration methods could shed light on the benefits of improved clearance of medium and high molecular weight solutes compared to hemodialysis. In any case, further studies are needed to assess and differentiate precisely factors contributing to pruritus of both origins.
